# Flexible high dielectric thin films based on cellulose nanofibrils and acid oxidized multi-walled carbon nanotubes

**DOI:** 10.1039/c9ra10915c

**Published:** 2020-03-16

**Authors:** Jie Tao, Shun-an Cao

**Affiliations:** School of Power and Mechanical Engineering, Wuhan University Wuhan 430072 China shunancao@163.com

## Abstract

Flexible high dielectric materials are of prime importance for advanced portable, foldable and wearable devices. A series of flexible high dielectric thin films based on cellulose nanofibrils (CNF) and acid oxidized multi-walled carbon nanotubes (*o*-MWCNT) was prepared in aqueous solution. Though no organic solvent was involved during the preparation, the SEM images showed that *o*-MWCNTs have good distribution within the CNF matrix. The dielectric constant of CNF/*o*-MWCNT (6.2 wt%) composite films was greatly increased from 25.24 for pure CNF to 73.88, while the loss tangent slightly decreased from 0.70 to 0.68, and the AC conductivity decreased from 3.15 × 10^−7^ S cm^−1^ for CNF to 1.77 × 10^−7^ S cm^−1^ (at 1 kHz). The abnormal decrease of loss tangent and AC conductivity were attributed to the introduction of oxide-containing groups on the surface of MWCNTs. The nanocomposite films showed excellent flexibility such that they could be bent a thousand times without visible damage. The presence of MWCNTs also helped to improve the thermal stability of the composite films. The excellent dielectric and mechanical properties of the CNF/*o*-MWCNT composite film demonstrate its great potential to be utilized in the field of energy storage.

## Abstract 2

The acid oxidized MWCNTs have excellent dispersity in CNF, resulting in outstanding dielectric properties of the flexible CNF/*o*-MWCNT nanocomposite films.

## Graphical abstract

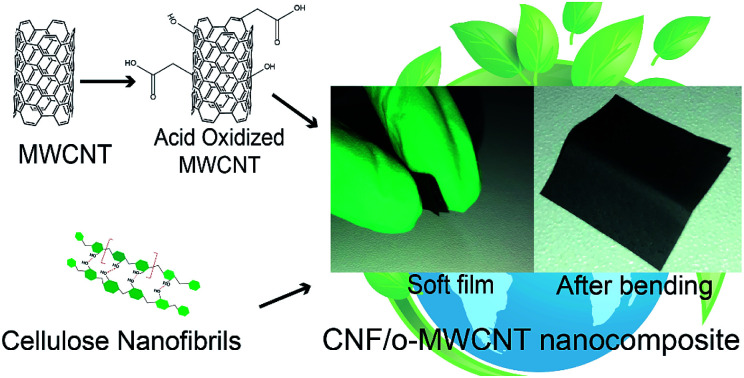

## Introduction

Nowadays, multifunctional materials have become the key to breakthroughs in many fields. Among them are soft high dielectric materials, which are in hugely demand in high-tech applications such as foldable touch screens, antennae, biosensors, inverters, organic transistors, and hybrid electrical vehicles.^1–3^ Materials with a high dielectric constant can considerably increase the energy density and power, accelerate the charge/discharge speed, and reduce the size of devices. Soft high dielectric materials are more preferred in portable, foldable and wearable electronic devices.^4^ Recently, nanocellulose has been proposed to be a replacement for traditional high dielectric polymer matrices, for their high dielectric constant, excellent mechanical properties, high transparency, lightweight and low coefficient thermal expansion.^5–11^ More attractively, cellulose is an almost inexhaustible bioresource, and nanocellulose can be prepared simply by mechanical grinding without toxic solvent, meeting the request for sustainable development.

Commonly used fillers for nanocellulose based high dielectrics are ceramic fillers (*e.g.*, barium titanate,^12^ titanate dioxide^13^) and conductive fillers (*e.g.*, silver nanowires,^3,14^ graphene,^15–18^ triglycine sulfate (TGS),^19–22^ polyaniline (PANI),^23,24^ carbon nanotubes^25^). High dielectric nanocellulose/ceramic composites usually demand high filler loading (>50 wt%), leading to severe agglomerations, poor mechanical properties, and low breakdown strength.^26^ On the contrary, a small addition of conductive filler can bring in much higher dielectric constant. According to the percolation theory,^27,28^ the dielectric constant grows dramatically near the threshold, which is usually around 5% for raw fillers and differs after different treatments. Beeran *et al.*^18^ used ammonia-functionalized graphene oxide (NGO) nanoplatelets as the filler and incorporated it into CNF and 2,2,6,6-tetramethylpiperidine-1-oxyl oxidized cellulose (TCNF), respectively. With an NGO loading of 3 wt%, the relative dielectric constant (*ε*_r_) of NGO/CNF (thickness 50 ± 2 μm) and NGO/TCNF (thickness 70 ± 2 μm) films are 50 and 158 (at 1 kHz), respectively. Inui *et al.*^14^ prepared a high-dielectric nanocomposite (thickness 50 μm) based on CNF and conductive silver nanowires (AgNWs, diameter ≈ 100 nm, length ≈ 10 μm). A small addition of AgNWs (2.54 vol%) results in an extremely high *ε*_r_ of 726.5 with a loss tangent (tan *δ*) of 0.26, while the *ε*_r_ of pure CNF is 5.3 with a tan *δ* of 0.2 (at 1.1 GHz). Anju and Narayanankutty^24^ coated CNF with conductive polyaniline (PANI) by an *in situ* polymerization technique in an aqueous medium and mixed the PANI/CNF filler with polyvinyl alcohol (PVA). The *ε*_r_ of the PVA/PANI/CNF composite reaches 4759 with a tan *δ* of 12 (at 1 kHz) near the percolation threshold (20 wt%). Zeng *et al.*^25^ employed TCNF as the matrix and multi-walled carbon nanotubes (MWCNT) as fillers. Three types of MWCNT fillers with different aspect ratios were compared. The results show that MWCNTs with a high aspect ratio have a larger dipole moment and lower percolation threshold, leading to higher *ε*_r_. The maximum *ε*_r_ of 3198 (at 1 kHz) with a tan *δ* of about 0.9 is obtained at a low CNT loading of 4.5 wt% (diameter < 8 nm), while the *ε*_r_ of pure TCNF is 15 with a tan *δ* of about 0.5.

The increase of *ε*_r_ is always accompanied by much-amplified tan *δ*, which is the ratio of dissipated energy and stored energy in each period when sine-wave voltage is applied. The dissipated energy converts to heat during polarization and depolarization process, leading to high working temperature, accelerated deterioration, and early material failure. Thus, many kinds of research have been made to achieve high *ε*_r_ while maintaining low tan *δ*. It is reported that the interface between the filler and the matrix is of vital importance.^29^ With improved compatibilities between the filler and the matrix, the filler can distribute more uniformly in the composite. The effective methods include applying ultrasonication, modifying the surface of fillers, and preparing matrix by *in situ* polymerizations. However, in most cases, complicated chemical reagents and treatments are involved.^15,18,30,31^

In this study, a series of high dielectric thin films with relatively low tan *δ* was prepared based on MWCNT and CNF. MWCNTs have excellent conductivity, large surface area and high aspect ratio, which are beneficial to superior dielectric properties of composites. Their one-dimensional structure makes it easy for them to align with each other and form a network at low loading, resulting in better mechanical properties. In addition, the dispersibility of MWCNT in aqueous can be greatly improved by simply acid oxidation, owing to the introducing of hydrophilic groups (*e.g.*, hydroxyl group, carboxyl group).^32,33^ Thus, CNF and MWVNT can be homogeneously mixed without organic solvent. Our previous study shows that CNF is a better choice than TCNF, considering its lower loss tangent and higher energy efficiency.^13^ Thus, CNF was used as the matrix. The results show that acid oxidized MWCNTs (*o*-MWCNTs) have good distribution in the CNF matrix, and the CNF/*o*-MWCNT exhibits excellent dielectric properties and thermal stabilities.

## Experimental

### Materials

Cellulose nanofibrils slurry (CNF, 3 wt% in water) was purchased from Cellulose Lab Inc. (Canada). The CNF has a diameter of 6–80 nm with a length of several micrometers.^34–37^ Multi-walled carbon nanotube (MWCNT, diameter < 10 nm, length 5–15 μm) was purchased from TCI Co., Ltd. (Shanghai, China). Nitric Acid (HNO_3_, AR, 65.0–68.0%) was supplied by Sinopharm Chemical Reagent Co., Ltd (Shanghai, China) and used as received. Deionized water was obtained by a Millipore Direct-Q3 water purification system (USA).

### Oxidation of MWCNT

When mixed directly with CNF, MWCNTs tended to aggregate with each other, form into large particles, and deposit on the bottom of the beaker. Therefore, MWCNTs were oxidized by concentrated HNO_3_ to improve their dispersity in water. In a typical experiment, 0.2 g of MWCNT was added into 20 mL of concentrated HNO_3_, and the mixture was subjected to an ultrasonic bath for 30 min. Subsequently, the mixture was stirred vigorously while heated up to 120 °C for 6 h. After being cooled to room temperature, the mixture was diluted with deionized water and centrifuged at 8000 rpm for 10 min. Then the supernatant was removed. The dilution and centrifugation processes were repeated until the pH value of the supernatant turned nearly neutral. Finally, *o*-MWCNT was obtained by drying the black sediment at 70 °C for about 12 h.

### Preparation of MWCNT/CNT nanocomposite films

**Fig. 1:**
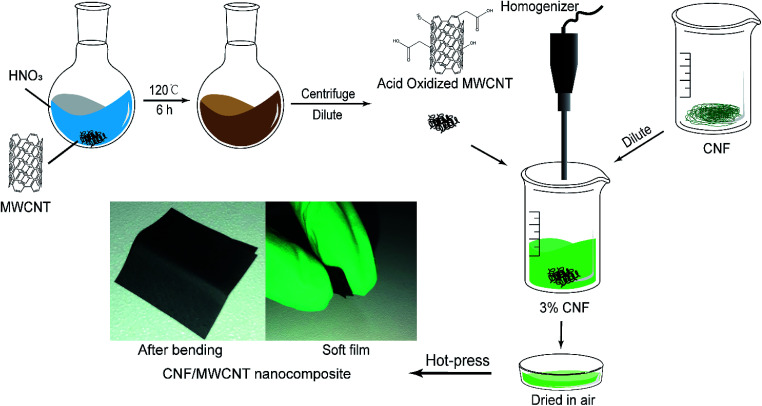
Preparation of CNF/*o*-MWCNT composite films.

The purchased CNF slurry was diluted into 0.3 wt% with deionized water and was stirred by a homogenizer (RCD-1A, Changzhouyuexin, China) for 5 min. After the dilution, a certain amount of *o*-MWCNT was added according to the dry solid content. Then the mixture was subjected to an ultrasonic bath for 30 min and homogenized for 5 min again. CNF have good dispersity in water owing to its abundant hydroxyl groups. And the oxidation of MWCNTs greatly improves their dispersity in water. Thus, these two components could be mixed well in water, and no organic solvent is needed. After being poured into a Petri dish, the mixture was dried in a fume hood for several days. Finally, the obtained films were hot-pressed under 80 °C for 3 hours. The thickness of the flexible films was in the range of 60–90 μm. The schematic of the preparation of CNF/*o*-MWCNT films is shown in Fig. 1. The paperlike films with a size of 2 cm × 2 cm were bent manually to make the top edge meet the bottom edge and then released. After repeating the process for a thousand times, no visible change was made (as shown in Fig. 1), which demonstrates its flexibility over other fragile or stiff dielectric composites.

### Measurements and characterization

The surface and cross-section morphologies of sample films were studied by field-emission scanning electron microscopy (FE-SEM, TESCAN MIRA3, Czech Republic) with an accelerating voltage of 5 kHz. Prior to the measurement, the samples were coated with a layer of gold using a sputter coater (Quorum SC7620, UK) in a vacuum to reduce charge interruptions. X-ray diffraction (XRD) patterns of the sample films were determined by an X-ray diffraction spectrometer (XRD, PANalytical XPert Pro, Netherlands) with Cu K_α_ radiation (*λ* = 1.542 Å) at 40 kV and 40 mA in the 2 theta (2*θ*) value range from 5° to 80°. Fourier Transform Infrared (FTIR) spectra of MWCNTs and CNF/*o*-MWCNT composite films were recorded with an FTIR spectrophotometer (Nicolet Impact-5700, USA) at a resolution of 2 cm^−1^ within the range of 4000–400 cm^−1^. The thermal stabilities of the nanocomposites were tested by thermogravimetric analysis and differential scanning calorimetry (TGA-DSC, Netzsch STA449F3, Germany) at a heating rate of 10°C min^−1^ from 35 °C to 790 °C under a nitrogen atmosphere. The dielectric constant, loss tangent and AC electrical conductivity of sample films were measured using an LCR meter (Keysight E4980A, USA). The results were recorded in the frequency range from 40 Hz to 1 MHz with an oscillation signal of 1 V at ambient temperature. Prior to the measurement, gold electrodes were sputtered on both sides of the specimens, and the test was repeated four times for each composition. The relative dielectric constants (*ε*_r_) of the sample films were calculated by eqn (1):1
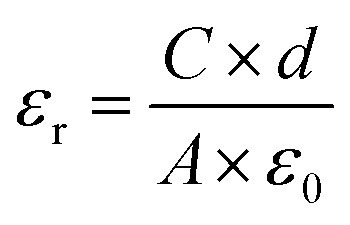
where, *C* is the capacitance; *ε*_0_ is the absolute dielectric constant of vacuum, *ε*_0_ = 8.854 × 10^−12^ F m^−1^; *A* is the electrode area, *A* = 4.52 × 10^−6^ m^2^; *d* is the thickness of the sample film. The polarization–electric field (P–E) curves of the sample films, with a ferroelectric tester (Radiant Precision Multiferroic II, USA) at 100 Hz.

## Results & discussion

### Morphology

**Fig. 2:**
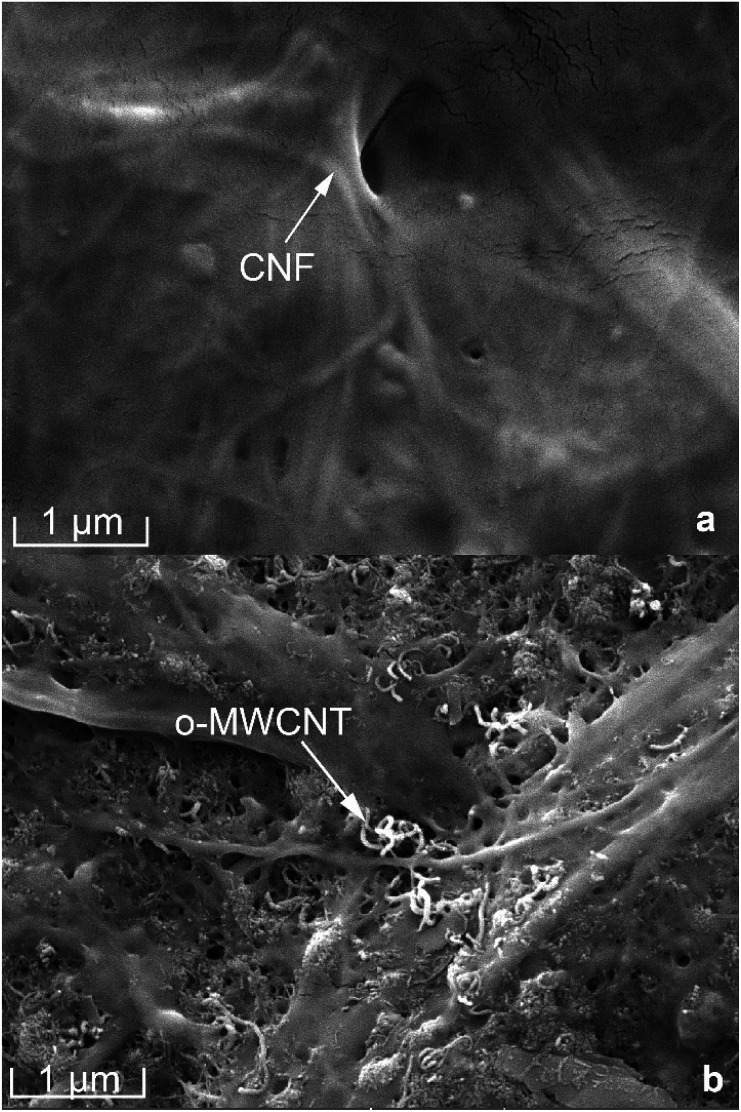
Surface FE-SEM micrographs of (a) pure CNF and (b) CNF/*o*-MWCNT (6.2 wt%) composite films.

The surface images of the pure CNF and CNF/*o*-MWCNT (6.2 wt%) composite films were investigated by FE-SEM (as shown in Fig. 2). Pure CNF film was compact and had a smooth surface (as shown in Fig. 2(a)). CNF/MWCNT composite films showed distinctly different morphologies. After the addition of *o*-MWCNT, the surface of the film became rough, and many pores were observed, revealing a loose film structure. The *o*-MWCNTs were uniformly dispersed throughout the CNF matrix when the filler content is lower than 6.2 wt%. With the further addition of *o*-MWCNTs, many bigger *o*-MWCNTs firstly aggregated with each other and formed small clusters. Then these clusters started to connect with each other and formed a network. This result shows that uniform CNF/*o*-MWCNT nanocomposite films can be obtained by simple mechanical mixing and casting methods when filler content below 6.2 wt%.

### Acid oxidation of MWCNT

**Fig. 3:**
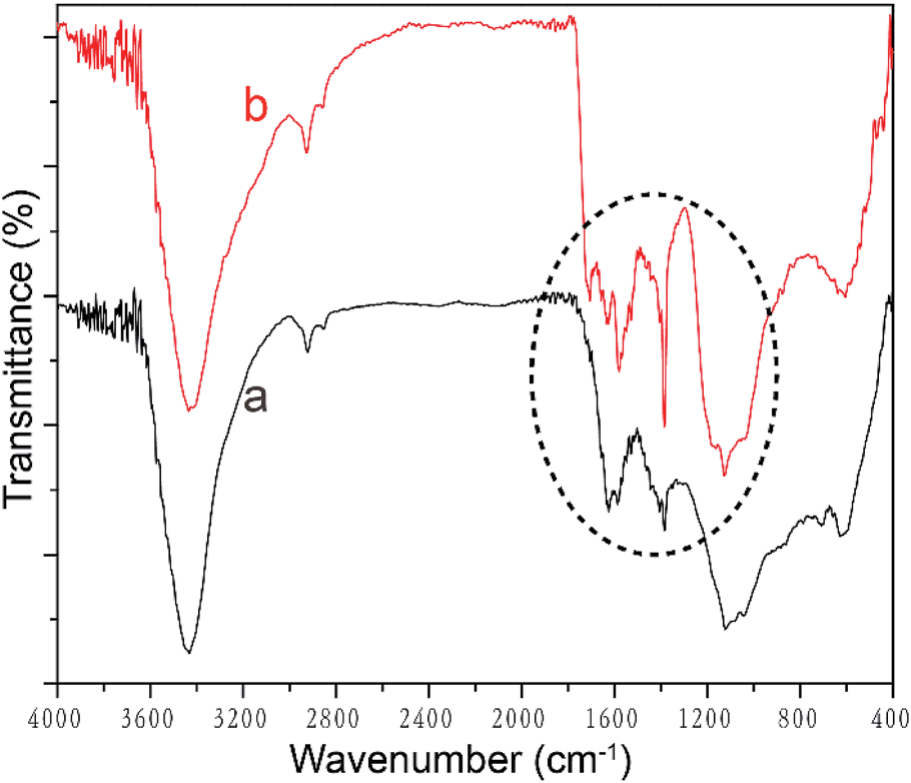
FTIR spectra of (a) pristine MWCNT and (b) acid oxidized MWCNT.

Acid oxidation is conducive to the dispersion of MWCNTs in water due to the introduction of hydrophilic groups, *e.g.*, hydroxyl and carboxyl groups. FTIR spectra were analyzed to identify these molecular structural differences of MWCNTs before and after the acid oxidation, as shown in Fig. 3. The FTIR spectrum of pristine MWCNT displays not only C=C peaks at 1383 cm^−1^ and 1122 cm^−1^, but also many other small peaks, namely, O–H peak at 3434 cm^−1^, C–H peaks at 2925 cm^−1^ and 2850 cm^−1^, and C=O peak at 1624 cm^−1^, indicating the presence of hydroxyl and carboxyl groups.^38–40^ These hydroxyl and carboxyl groups in as-received MWCNT could be attributed to the purification process by the manufacturer.^41^ On acid treatment, two characteristic peaks of the carboxyl group are seen at 1705 cm^−1^ and 1579 cm^−1^, respectively (as shown in the dashed circle of Fig. 3), demonstrating that more COOH groups are introduced by the acid oxidation.^39^ Additionally, the peaks at 1624 cm^−1^, 1383 cm^−1^ and 1122 cm^−1^ shifts to 1633 cm^−1^, 1384 cm^−1^, and 1125 cm^−1^, respectively, it also reveals the structure changes of MWCNT upon carboxylation.

### Dielectric properties

**Fig. 4:**
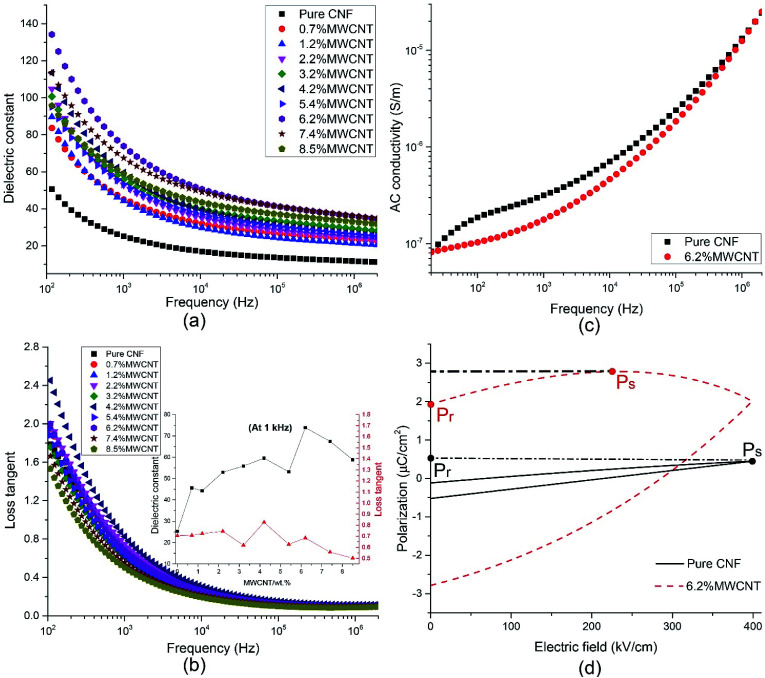
The influence of frequency and filler content on the (a) dielectric constant, (b) loss tangent, and (c) AC conductivity. (d) P–E hysteresis curves of CNF/*o*-MWCNT nanocomposites.

The influence of filler loading and frequency on dielectric constant (*ε*_r_) and loss tangent (tan *δ*) were studied, as presented in Fig. 4(a) and (b). In the lower frequency range of 40–10 kHz, both *ε*_r_ and tan *δ* decrease rapidly with the increase of frequency. It is ascribed to the electrode effect and Maxwell–Wagner–Sillars interfacial polarization.^24,31,42–44^ When an electric field is applied on the nanocomposite film, many dipoles and charge carriers accumulate at the interface between the electrode and the sample and between the fillers and matrices. With the frequency going up, the high periodic reversal of the electric field makes it harder for the dipoles and charge carriers to catch up with the change of frequency. Hence, the interfacial polarization is declined and results in lower *ε*_r_ and tan *δ*.^45^ In higher frequency range, both *ε*_r_ and tan *δ* tend to remain unchanged, which demonstrates that electronic polarization, atomic polarization, and orientation polarization starts to replace interfacial polarization to play a predominant role.

The filler loading shows more effects on dielectric properties in the lower frequency range of 40 Hz to 10 kHz. The *ε*_r_ rises with the addition of MWCNT when the filler content is lower than 6.2 wt% and falls with the further addition of MWCNT. In the meantime, the tan *δ* fluctuates on a narrow scale. It can be explained by percolation theory,^27,28,46^ according to which, with the addition of conductive filler, the filler particles firstly aggregate with each other, then form conductive clusters. When the filler content is close to the percolation threshold, the conductive clusters start to connect with each other and result in a conductive path. At the same time, the insulative composite turns into a conductor. The dielectric constant and conductivity of composite obey the following the power law equation.^26,47–51^

2 *ε* ∝ *ε*_m_(*ϕ*_c_ − *ϕ*_f_)^−*s*^, *ϕ*_f_ < *ϕ*_c_

3 *σ* ∝ *σ*_m_(*ϕ*_c_ − *ϕ*_f_)^−*s*^, *ϕ*_f_ < *ϕ*_c_

4 *σ* ∝ *σ*_f_^*u*^*σ*_m_^1−*u*^, |*ϕ*_f_ − *ϕ*_c_| → 0

5 *σ* ∝ *σ*_f_(*ϕ*_f_ − *ϕ*_c_)^*t*^, *ϕ*_f_ > *ϕ*_c_

where, *ϕ*_f_ is the volume fraction of filler, *ϕ*_c_ is the volume fraction at the percolation threshold, *σ*_m_ and *σ*_f_ are the electrical conductivities of the polymer matrix and the conductive fillers, respectively. *s* and *t* are the critical exponents, and *u* = *t*/(*t* + *s*). In this case, the percolation threshold is 6.2 wt%. It is noticeable that the tan *δ* remains nearly unchanged, and it is attributed to the functional groups at the surface of MWCNTs. The *ε*_r_ of CNF/MWCNT composite films reaches its maximum value of 73.88 with 6.2 wt% of MWCNT. It is about three times higher than that of pure CNF (25.24, at 1 kHz). Meanwhile, the tan *δ* slightly decrease from 0.70 to 0.68. The outstanding dielectric properties of CNF/*o*-MWCNT nanocomposite film make it a promising candidate for capacitor applications.

To further explore the electric properties of CNF/MWCNT (6.2 wt%) nanocomposite, the frequency dependence of AC conductivity (*σ*_AC_) was tested and compared with pure CNF (as shown in Fig. 4(c)). The *σ*_AC_ of both samples go up with the increase of frequency. In the lower frequency range of 40 Hz to 100 kHz, the trend was gradual, while it turned rapidly with the frequency further increased. This phenomenon complies well with Dyre's Random Free Energy Barrier Model.^52^ The frequency-dependent property of *σ*_AC_ is related to the hopping of the charge carriers in the localized state and upper states in the conduction band. And this process can be accelerated by higher frequency.^12,53^

Interestingly, the composite film with 6.2 wt% of *o*-MWCNT exhibits lower *σ*_AC_ than that of pure CNF. At 1 kHz, the *σ*_AC_ decreases from 3.15 × 10^−7^ S cm^−1^ for CNF to 1.77 × 10^−7^ S cm^−1^ for CNF/MWCNT (6.2 wt%). Cellulose contains many hydrophilic hydroxyl groups, resulting in absorbed moisture and increased conductivity. The addition of *o*-MWCNTs brings in many oxide-containing groups (*e.g.* carboxyl groups), which are more attractive to the hydroxyl groups in CNF to form hydrogen bonds than water molecules, leading to lower absorbed moisture and decreased conductivity.

Polarization–electric field (P–E) hysteresis curve is an efficient way to evaluate the energy storage properties. The P–E hysteresis curves of pure CNF and the CNF/*o*-MWCNT (6.2 wt%) were measured, as shown in Fig. 4(d). Both remnant polarization (*P*_r_) and saturated polarization (*P*_s_) of the CNF/MWCNT nanocomposite film are higher than that of pure CNF. It demonstrates that the addition of *o*-MWCNT makes it more easily for samples to be polarized, which is also proved by the results of higher *ε*_r_ of CNF/*o*-MWCNT composites than that of pure CNF. However, the *P*_r_ of the composite film is close to *P*_s_, suggesting a low efficiency of energy storage.^12^ The polarization of the CNF/MWCNT films saturates before the electric field reaches its maximum value. It may be caused by the leakage current, which is the result of a heterogeneous structure and partial conductive network. With more efforts made on improving the dispersion of MWCNT fillers and reducing the remnant polarization, the CNF/MWCNT nanocomposite film has great potential in energy storage applications.

### Thermal stability

**Fig. 5:**
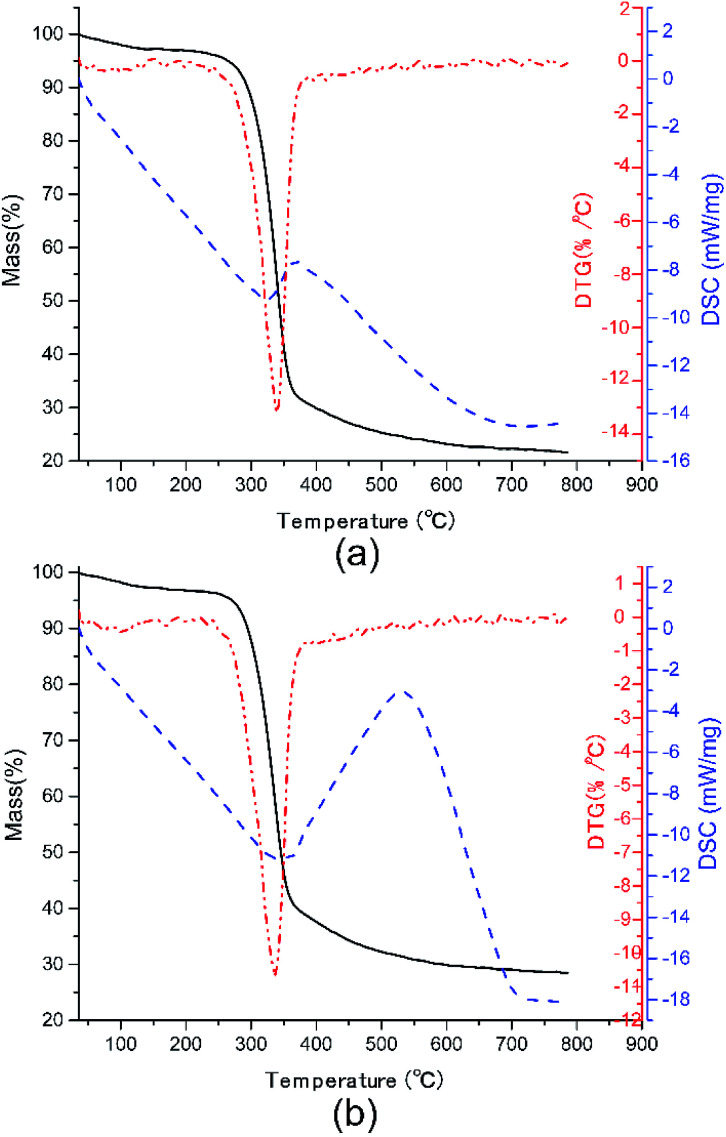
TGA-DSC curves of (a) pure CNF and (b) CNF/*o*-MWCNT (6.2 wt%) nanocomposite film.

In practice, the life length and reliability of dielectric materials are closely bound up with their thermal stability. Thus, the TGA-DSC curves of pure CNF and CNF/*o*-MWCNT (6.2 wt%) composite film were measured, as depicted in Fig. 5. Two stages are presented in the degradation process. The first degradation stage is the slight weight loss before 100 °C, resulting from the evaporation of absorbed water.^13^ The weight loss of CNF/*o*-MWCNT (6.2 wt%) slightly decreased from 2.02% to 1.82%. It indicates that the introduction of oxide-containing groups helps to decrease absorbed moisture. The temperature at 5.0% weight loss (*T*_5%_) is used as the start signal of degradation. CNF/*o*-MWCNT (6.2 wt%) nanocomposite has a *T*_5%_ of 271 °C, which was a little higher than that of pure CNF (265 °C). It means that the incorporation of MWCNTs helps to improve thermal stability. The second degradation stage is in the temperature range from 250 °C to 400 °C, where there is a sharp drop of weight, as well as a DTG peak at 340 °C and a DSC endothermal peak at 325 °C. It is due to the decomposition process of CNF. With the addition of MWCNTs, the DTG peak shifted slightly from 340 °C to 343 °C, and the DSC endothermal peak shifted from 325 °C to 341 °C. It also illustrates that the incorporation of MWCNTs helps to improve thermal stability. In a word, the TGA-DSC curves suggest that the CNF/MWCNT composite films have acceptable water absorption and good thermostability.

## Conclusions

A series of flexible high dielectric nanocomposite films based on CNF and acid oxidized MWCNT were prepared by casting method in aqueous solution. Though no organic solvents were involved during the preparation, MWCNT had good distribution within the CNF matrix. The porous structure appears with the addition of *o*-MWCNT, and its adverse effects are reflected in the dielectric properties. The dielectric constant of CNF/*o*-MWCNT (6.2 wt%) composite films greatly increases from 25.24 for pure CNF to 73.88, while the loss tangent slightly decreases from 0.70 to 0.68, and the AC conductivity decreases from 3.15 × 10^−7^ S cm^−1^ for CNF to 1.77 × 10^−7^ S cm^−1^ (at 1 kHz). The abnormal decrease of loss tangent and AC conductivity are attributed to the introduction of oxide-containing groups on the surface of MWCNTs. The nanocomposite films show excellent flexibility that they can be bent for a thousand times without obvious damage. The presence of MWCNT helps to improve the thermal stability of the composite films. With more efforts being made to cut down the remanent polarization and improve the compatibility between the MWCNT filler and CNF matrix, the novel CNF/MWCNT composite films is a promising green candidate for high dielectrics in the field of energy storage.

## Conflicts of interest

There are no conflicts to declare.

## Supplementary Material
